# Evaluating Human Papillomavirus eHealth in Hmong Adolescents to Promote Vaccinations: Pilot Feasibility Study

**DOI:** 10.2196/38388

**Published:** 2023-06-20

**Authors:** Hee Yun Lee, Serena Xiong, Aparajita Sur, Tounhia Khang, Bai Vue, Kathleen A Culhane-Pera, Shannon Pergament, M Beatriz Torres, Joseph S Koopmeiners, Jay Desai

**Affiliations:** 1 School of Social Work The University of Alabama Tuscaloosa, AL United States; 2 School of Medicine Washington University in St Louis St Louis, MO United States; 3 School of Public Health University of Minnesota Twin Cities, MN United States; 4 SoLaHmo Partnership for Health & Wellness Community University Health Care Center Minneapolis, MN United States; 5 Department of Public Health Mercyhurst University Erie, PA United States; 6 Minnesota Department of Health Saint Paul, MN United States

**Keywords:** human papillomavirus, HPV, HPV vaccine, community-based participatory action research, CBPAR, eHealth, website, social cognitive theory, Hmong, mobile phone

## Abstract

**Background:**

Human papillomavirus (HPV) is a common sexually transmitted infection, causing multiple cancers, including cervical, penile, and anal. Infection and subsequent health risks caused by HPV can be diminished by HPV vaccination. Unfortunately, vaccination rates among Hmong Americans are substantially lower than those among other racial and ethnic groups, despite having higher cervical cancer rates than non-Hispanic White women. Such disparities and sparse literature highlight the need for innovative and culturally appropriate educational interventions to improve HPV vaccine rates in Hmong Americans.

**Objective:**

We aimed to develop and evaluate the effectiveness and usability of an innovative web-based eHealth educational website, the *Hmong Promoting Vaccines* website (HmongHPV website), for Hmong-American parents and adolescents to improve their knowledge, self-efficacy, and decision-making capacities to obtain HPV vaccinations.

**Methods:**

Through social cognitive theory and community-based participatory action research process, we created a theory-driven and culturally and linguistically appropriate website for Hmong parents and adolescents. We conducted a pre-post intervention pilot study to assess the website’s effectiveness and usability. Overall, 30 Hmong-American parent and adolescent dyads responded to questions about HPV and HPV vaccine knowledge, self-efficacy, and decision-making at preintervention, 1 week after intervention, and at the 5-week follow-up. Participants responded to survey questions about website content and processes at 1 and 5 weeks, and a subset of 20 dyad participants participated in telephone interviews 6 weeks later. We used paired *t* tests (2-tailed) to measure the change in knowledge, self-efficacy, and decision-making processes, and used template analysis to identify a priori themes for website usability.

**Results:**

Participants’ HPV and HPV vaccine knowledge improved significantly from pre- to postintervention stage and follow-up. Knowledge scores increased from preintervention to 1 week after intervention for both parents (HPV knowledge, *P*=.01; vaccine knowledge, *P*=.01) and children (HPV knowledge, *P*=.01; vaccine knowledge, *P*<.001), which were sustained at the 5-week follow-up. Parents’ average self-efficacy score increased from 21.6 at baseline to 23.9 (*P=*.007) at post intervention and 23.5 (*P*=.054) at follow-up. Similar improvements were observed in the teenagers’ self-efficacy scores (from 30.3 at baseline to 35.6, *P*=.009, at post intervention and 35.9, *P*=.006, at follow-up). Collaborative decision-making between parents and adolescents improved immediately after using the website (*P*=.002) and at follow-up (*P*=.02). The interview data also revealed that the website’s content was informative and engaging; in particular, participants enjoyed the web-based quizzes and vaccine reminders.

**Conclusions:**

This theory-driven, community-based participatory action research–designed and culturally and linguistically appropriate educational website was well received. It improved Hmong parents’ and adolescents’ knowledge, self-efficacy, and decision-making processes regarding HPV vaccination. Future studies should examine the website’s impact on HPV vaccine uptake and its potential for broader use across various settings (eg, clinics and schools).

## Introduction

### Background

Nearly 80 million men and women in the United States are infected by the human papillomavirus (HPV), making it the most common sexually transmitted infection [1]. HPV is responsible for 75% of vaginal, 69% of vulvar, 63% of penile, 91% of anal, and 72% of oropharyngeal cancers [2-4]. Cervical cancer is a serious public health threat for Hmong-American women, who have cervical cancer diagnosis rates over 4 times more than those of non-Hispanic White Americans and 3 times than those of other Asian-American women (eg, Chinese, Vietnamese, Cambodian, and Korean) [5].

Uptake of HPV vaccines can reduce the occurrence of such cancers. However, HPV vaccination rates are alarmingly low in all adolescents. Numerous studies have shown that Asian-American populations have even lower HPV vaccination rates [6-10]. According to data collected by the United States Department of Health and Human Services, only 60% of adolescent Asian-American female individuals and 41% of male individuals have completed 2 or 3 HPV vaccine doses, respectively [11,12]. Although the literature on HPV vaccination rates in Hmong-American youth does not exist, a 2015 medical record review of Hmong-American youth aged 9 to 17 years at a Minnesota community health center found HPV vaccination completion rates of 32% for girls and 20% for boys, much lower than the national rates of 57% and 35%, respectively [13]. This disparity indicates that strategies to support and foster HPV vaccination are urgently needed in the Hmong-American community.

One potential reason for the limited uptake of the HPV vaccine in Hmong Americans may be the low parental and adolescent awareness and knowledge of HPV effects and HPV vaccine benefits. In a conveniently sampled survey of Hmong Americans, only 38% of boys had heard of the HPV vaccine [14]. In another study assessing the level of HPV knowledge among Hmong Americans, participants aged ≥18 years received an average score of 4.7 out of 7 on an HPV literacy measure [15].

Barriers to HPV knowledge and vaccination within the Hmong-American community exist at multiple levels [13]. Similar to other recent Asian-American immigrants, Hmong Americans face many individual and structural challenges, including poverty, language barriers, inadequate access to or use of health care, poor health literacy, and lack of transportation [16], which can affect HPV vaccine rates [15,17-22]. The Hmong-American population has the highest poverty rate among all Asian-American groups [20-22]. This, compounded with limited English proficiency and low literacy, makes Hmong Americans and other Asian Americans particularly vulnerable to underutilizing preventive health care [13,23], such as the uptake of HPV vaccinations [9,15].

### This Study

eHealth uses digital platforms such as dynamic websites, apps, or mobile and wireless devices to deliver health information and engage users to improve health literacy, behaviors, and outcomes [24]. eHealth is emerging as a direct and effective medium to modify health behavior, especially during the COVID-19 pandemic [25-27]**.** Over 90% of adults in the United States have internet access, 96% have some type of cell phone, and these trends continue to increase [28]. According to Nielsen ratings, 97% of Asian Americans have smartphones [29]. The proliferation of cell phone ownership provides an ideal environment for building on existing eHealth interventions proven to increase HPV vaccination uptake [30,31]. eHealth approaches that can be culturally and personally tailored using multilevel and multimedia approaches may be ideal for engaging Hmong parents and adolescents in HPV vaccination.

Health behavior change, such as initiating HPV vaccinations, is a complex process influenced by many factors. Using a relevant theory for behavior change, such as Social Cognitive Theory (SCT), can guide the creation of an effective intervention targeted at multiple factors (eg, environment and individual) [32]. Engaging community members in finding solutions to community health problems, such as a community-based participatory action research (CBPAR) approach, can increase the acceptability and usability of interventions [33,34]. Using an SCT-based and CBPAR-designed pilot feasibility study approach, this study aimed to develop and evaluate a culturally and linguistically appropriate educational website called *Hmong Promoting Vaccines* (HmongHPV website) to improve their knowledge, self-efficacy, and decision-making capacities to obtain HPV vaccinations among Hmong-American parents and adolescents.

## Methods

### Research Approach: CBPAR

We used the CBPAR approach to develop and evaluate an innovative, web-based eHealth educational website. CBPAR is a research approach in which community members, academicians, and providers form an egalitarian partnership to conduct the study, including the formulation of research questions, intervention design, recruitment, implementation, data analysis, and dissemination of research findings [33,34]. The benefits of CBPAR include increasing community trust in research, appropriately tailoring the intervention, increasing the external validity of research findings, improving the likelihood that research results will lead to effective programs and products that are useful to communities, and creating stronger connections between stakeholders [33,34]. Our CBPAR team consisted of 5 Hmong academic community members and 7 non-Hmong academicians. Additional Hmong community guidance was provided by a community advisory board (CAB) consisting of Hmong-American parents, community leaders, nurses, and a family physician. The study team worked with the CAB members in developing focus group questions for parents and youth, creating educational content and website design, developing the pre-post evaluation questions, recruiting study participants, interpreting qualitative and quantitative results, and providing feedback for future dissemination of the educational website [13].

### Development of the HmongHPV Website

SCT guided the development of the web-based educational HmongHPV website [32,35]. SCT relies on the dynamic relationship between personal, behavioral, and environmental factors and how these factors influence thoughts and actions [32,35]. Therefore, SCT is an essential component for improving knowledge, health behaviors, and self-efficacy [35]. On the basis of SCT principles, the educational components of the website were designed to impact participants’ cognitive factors, such as knowledge, self-efficacy, and decision-making processes.

Findings from focus groups with Hmong-American parents and adolescents shaped our educational intervention’s content and communication strategies [13]. Parents prefer gain-framed messages, so their messages focus on protecting their children from HPV-related cancers [36]. Adolescents prefer loss-framed messages, so their messages focus on minimizing their losses, including vaccine pain and HPV diseases [36]. Both Hmong-American parent and adolescent participants perceived messages from authority figures, such as doctors, teachers, and recognized community members, to be more persuasive and influential than other sources; therefore, messages were delivered by a variety of health care professionals and community leaders [36].

The HmongHPV educational modules were designed by our academic community research team, CAB members, and a Hmong-based media organization. HmongHPV is an internet-based website with (1) a 5-module educational program tailored to Hmong-American parents or teens; (2) links to HPV-related resources; (3) a GPS locator feature for nearby health care clinics to obtain an HPV vaccination; including potential financial support; and (4) automated text reminders to complete educational components and schedule clinic visits for recommended HPV vaccinations. In addition, a bilingual Hmong-American health navigator was available to support the users with additional questions. The 5 educational modules included didactic learning with written text, visual pictures, videos, and web-based quizzes based on SCT. The 5 topic areas were (1) HPV infection and diseases; (2) HPV vaccine; (3) communication between clinics, parents, and teens; (4) concerns about HPV vaccines; and (5) clinics to get HPV vaccines. Parents’ and adolescents’ intervention contents were similar, but the message strategy followed either the gain or loss frame. In addition, the parents’ messages were available in Hmong and English, whereas the adolescents’ messages were in English only. The modules ranged in length from 7 to 10 minutes to complete and were intended to be viewed 1 per day; however, our CAB recommended allowing users to view the modules based on their learning style or convenience within a 1-week time frame. Over 8 months, the research team members worked with website developers (housed in the Educational Technology Innovations group at the University of Minnesota) to draft and finalize the website, which underwent alpha and beta testing to optimize the website’s usability and acceptability with community members and the CAB.

### Study Design, Recruitment, and Data Collection

To evaluate the effectiveness and usability of the *Hmong HPV* website, we used a quasi-experimental, pre-post study design consisting of surveys and interviews. We recruited Hmong parents and adolescents to evaluate the HmongHPV website at Hmong schools, Hmong community events, and Hmong markets via in-person discussions and culturally and linguistically appropriate flyers on social media such as Facebook, listserves, and emails; at a local community clinic through calling Hmong patients between the ages of 12 and 16 years who had and had not had their HPV vaccines; and by word of mouth. With slow enrollment, we increased the study incentives from US $50 to US $150 per person.

After providing informed consent, participants completed the preintervention questions, reviewed the website’s 5 modules, and then completed postintervention questions 1 week later and follow-up questions 5 weeks later. A subset of adolescents and parents also completed telephone-based interviews to assess the website's usability. Table 1 provides an overview of the survey instruments, including questionnaire topics (ie, HPV and HPV vaccine knowledge, self-efficacy, and decision-making), citations, how each question is analyzed, and where the results are displayed.

**Table 1:** Description of survey instruments.

Survey topic (reference number)	Number of items	Who answered	Type of response	How item was adjusted for analysis
HPV^a^ knowledge [37]	14	Parents and adolescents	Numerical score (sum of correct responses)	No adjustment
HPV vaccine knowledge [37]	12	Parents and adolescents	Numerical score (sum of correct responses)	No adjustment
Concerns about HPV and HPV vaccine [38,39]	7	Parents and adolescents	Numerical 1-5 Likert scale (1=strongly disagree; 5=strongly agree)	Binary (4=agree or 5=strongly agree vs Likert scores 1-3)
Parents’ statements about adolescents getting HPV vaccine [38,40]	6	Parents	Categorical (agree, disagree, and not sure)	Binary (agree vs disagree or not sure)
Communicating and seeking information about HPV vaccines [38-40]	10	Parents and adolescents	Categorical (yes, no, and not sure)	Binary (yes vs no or not sure)
Parents’ decision-making [13]	5	Parents	Numerical 1-5 Likert scale; 1=strongly disagree; 5=strongly agree	Binary (4=agree or 5=strongly agree vs Likert scores 1-3)
Adolescents’ decision-making [39]	6	Adolescents	0-5 Likert scale; 1=strongly disagree; 5=strongly agree	Binary (4=agree or 5=strongly agree vs Likert scores 1-3)
Adolescent self-efficacy [40]	5	Adolescents	0-100 scale, with: 0=cannot do at all; 100=highly certain can do	Changed to 0-10 scale
Parent self-efficacy [39]	7	Parents	1-4 Likert scale: 1=very unsure; 4=very sure	Sum of Likert responses

^a^HPV: human papillomavirus.

### Ethics Approval

The University of Minnesota gave institutional review board approval for the implementation of the study. Participants were provided with US $150 gift cards to compensate for their time. The study data were anonymized and deidentified for confidentiality. All the procedures performed in this study were in accordance with the ethical standards of the University of Minnesota Institutional Review Board on Social and Behavioral Research (#1612S01841).

### Data Analysis

#### Quantitative Data Analysis

Composite scores assessing HPV disease and HPV vaccine knowledge were calculated as the number of correct responses to the 14 and 12 questions, respectively. “Not sure” answers were treated as incorrect responses for analysis. Adolescents answered 5 self-efficacy questions, with responses ranging from 0 (least confident) to 100 (most confident), which were rescaled from 0 to 10. Parents’ self-efficacy scores were the sum of seven 0-4 Likert responses. Other continuous measures such as decision-making regarding HPV vaccination and HmongHPV usability and acceptability were answered on a 0-5 Likert scale. The remaining outcomes were categorical with “Yes,” “No,” and “Not Sure” responses.

All analyses were performed using R (version 3.6.1; R Foundation for Statistical Computing) [41]. Our analysis focused on changes between preintervention and 1 week post intervention and between preintervention and 5-week follow-up. Differences in continuous measures from pre- to post intervention and preintervention to follow-up were analyzed using paired *t* tests (2-tailed) and summarized with means and 95% CIs. Changes from baseline for binary endpoints were analyzed using McNemar test if there were >25 discordant pairs or analyzed using the exact binomial test if there were <25 discordant pairs. Only univariate analyses were conducted because of the small sample size of the pilot study.

#### Qualitative Data Analysis

Template analysis was used to analyze the interview data [42]. The research team collaboratively created an a priori coding scheme based on PSSUQ (Poststudy System Usability Questionnaire) domains of information quality, interface quality, and system usefulness. Furthermore, 2 Hmong team members independently coded the interview data and organized the data using codes on a Microsoft Excel spreadsheet. After this initial coding, the entire research team examined the coding scheme, discussed disagreements regarding codes until a consensus was reached, and created a modified coding scheme. Coders recoded the data using the final coding tree, which the entire team reviewed to analyze and identify themes.

## Results

### Participant Characteristics

A total of 30 parent-adolescent dyads were enrolled in the study, and 29 pairs completed the pre-post questions. Of the 20 dyads invited to participate in the qualitative interviews, 19 dyads and 1 parent completed the telephone interviews (1 child declined to participate). Table 2 presents the sociodemographic characteristics of the 29 dyads. Nearly 70% (20/29) of the adolescents were aged <15 years and all reported English as their primary language. Key parental characteristics included being from Laos or Thailand (20/29, 68%), practicing animism or shamanism (17/29, 59%), attending college (16/29, 55%), reporting Hmong as their primary language (9/29, 31%), and reporting a household income of less than US $75,000 (19/29, 66%). State-funded insurance was reported by 28% (8/29) of parents, no insurance was reported by 10% (3/29), and 90% (26/29) reported a regular health care provider or clinic. Almost 70% (20/29) of the parents reported having a routine check-up in the last 12 months, whereas 79% (23/29) reported their child having a routine check-up. Only 34% (10/29) of the parents reported receiving influenza vaccination during the same period. Most parents and adolescents used cellular or wireless networks (27/29, 93% and 25/29, 86%, respectively), used smartphones or cell phones for internet access (27/29, 93% and 23/29, 79%), used home computers for internet access (27/29, 90% and 23/29, 79%), had tablets or iPads (Apple Inc; 20/29, 69% 14/29, 48%), and had an unlimited data sharing plan (21/20, 72% and 14/29, 48%, respectively). In addition, all mobile phone users have texting, photo, and video capabilities.

**Table 2:** Characteristics of the study participants.

Characteristics			Parents (n=29)		Adolescents (n=29)
Age (years), mean (range)			40 (31-53)		13 (11-17)
**Sex^a^, n (%)**					
	Female	22 (79)		15 (52)	
	Male	6 (21)		14 (48)	
**Religion^a^, n (%)**					
	Animism or shamanism	17 (59)		14 (50)	
	Christianity	8 (28)		3 (11)	
	None	3 (10)		8 (29)	
	Other	1 (3)		3 (11)	
**Marital status^a^, n (%)**					
	Married	20 (69)		N/A^b^	
	Single	4 (14)		27 (93)	
	Unmarried relationship	4 (14)		1 (4)	
	Divorced	1 (3)		N/A	
**Country of origin^a^, n (%)**					
	United States	9 (31)		27 (93)	
	Thailand	10 (34)		1 (4)	
	Laos	10 (34)		0	
**Primary language, n (%)**					
	English	20 (69)		29 (100)	
	Hmong (White)	7 (24)		0	
	Hmong (Green)	2 (7)		0	
**Parent’s employment status**					
	Employed for wages or self-employed	20 (69)		N/A	
	Unemployed, unable to work, or retired	5 (17)		N/A	
	Student	3 (10)		N/A	
	Homemaker	1 (3)		N/A	
**Parent’s education level**					
	Attended college	16 (55)		N/A	
	High school graduate	9 (31)		N/A	
	Did not attend school or did not finish high school	4 (14)		N/A	

^a^Missing data: 1 parent did not indicate sex, and 1 child did not answer questions about religion, marital status, or country of origin.

^b^N/A: not applicable.

### Knowledge of HPV and HPV Vaccination

At baseline, parents and adolescents demonstrated that they understood the value of vaccines in general but not the specifics of the HPV vaccine. Composite scores of HPV knowledge and HPV vaccine knowledge (Table 3) increased significantly from preintervention to 1 week post intervention for parents (HPV knowledge *P=*.001; HPV vaccine knowledge *P*<.001) and adolescents (HPV knowledge *P*<.001; HPV vaccine knowledge *P*<.001) and were sustained at the 5-week follow-up. The median correct responses to the 14 HPV knowledge questions increased from 6 to 11 for parents and from 4 to 10 for children. Of the 12 HPV vaccine knowledge questions, the median number of correct responses increased from 8 to 11 for parents and from 6 to 10 for children. Many of these improvements are attributable to initial “Not sure” responses shifting to correct answers. Multimedia Appendix 1 provides individual measurement findings.

**Table 3:** Change in parents and adolescents’ composite scores for human papillomavirus (HPV) knowledge, HPV vaccine knowledge, and self-efficacy.

Mean score (possible range)		Preapp, mean (SD)	Postapp, mean (SD)	Follow-up, mean (SD)	Pre vs post, mean (95% CI)	*P* value^a^	Pre vs follow-up, mean (95% CI)	*P* value^a^
**Parents**								
	HPV knowledge (0-14)	6.03 (4.01)	10.31 (3.07)	10.21 (3.05)	4.28^b^ (2.96-5.59)	<.001	4.17^c^ (2.67-5.67)	<.001
	HPV vaccine knowledge (0-12)	7.03 (3.80)	9.90 (2.24)	9.68 (2.79)	2.86^b^ (1.59-4.13)	<.001	2.66^b^ (1.46-3.85)	<.001
	Self-efficacy (4-28)	21.6 (4.67)	23.9 (4.08)	23.5 (4.69)	2.3^b^ (0.7-3.9)	.007	1.9 (0.03-3.83)	.054
**Adolescents**								
	HPV knowledge (0-14)	4.06 (3.52)	9.31 (2.74)	9.24 (2.60)	5.24^b^ (3.91-6.57)	<.001	5.17^c^ (4.01-6.33)	<.001
	HPV vaccine knowledge (0-12)	5.86 (3.42)	8.82 (2.79)	8.89 (3.12)	2.96^b^ (1.48-4.02)	<.001	3.0 ^b^ (1.61-4.46)	<.001
	Self-efficacy (0-50)	30.3 (12.9)	35.6 (11.9)	35.9 (10.6)	5.3^b^ (1.4-9.1)	.009	5.6^b^ (1.7-9.6)	.007

^a^All paired *t* tests (2-tailed) were 2 sided. The differences in mean scores pre- and post-HPV app and follow-up are shown with their 95% CI and *P* values.

^b^*P*<.01.

^c^*P*<.05.

### Concerns About HPV and HPV Vaccination

**Figure 1:**
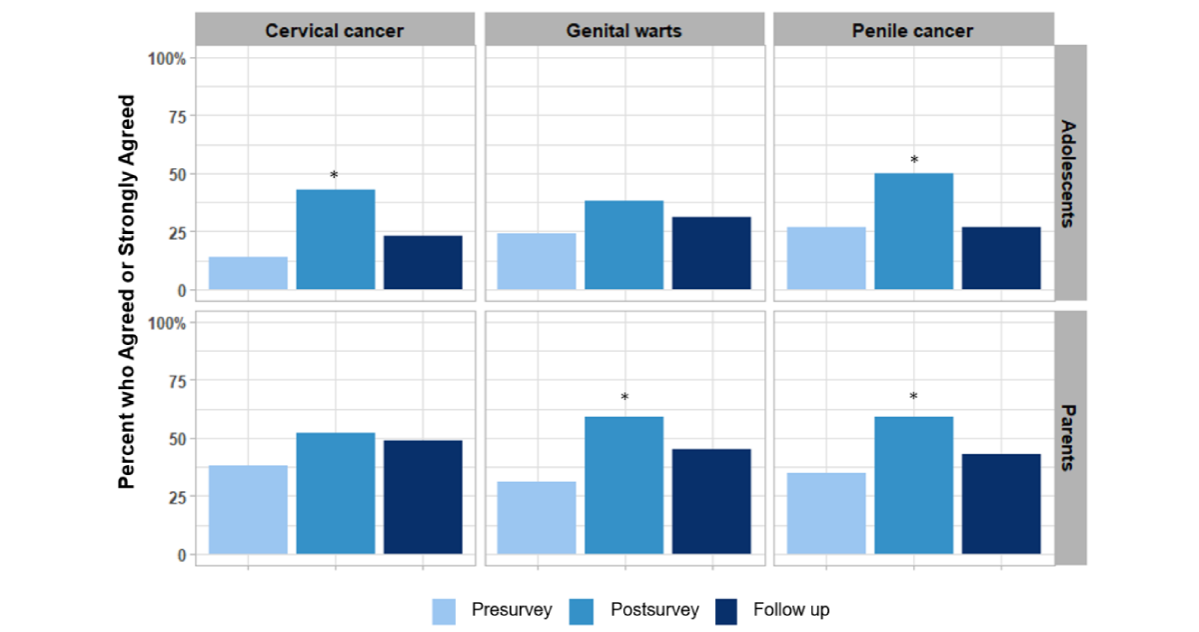
Concerns about the impact of HPV on cancer and genital warts. For adolescents, only female responses were depicted for cervical cancer concerns, and only male responses were depicted for penile cancer concerns. McNemar test assessed differences in pre- versus post app and pre- versus follow-up app. ** *P*<.01, * *P*<.05. HPV: human papilloma virus.

**Figure 2:**
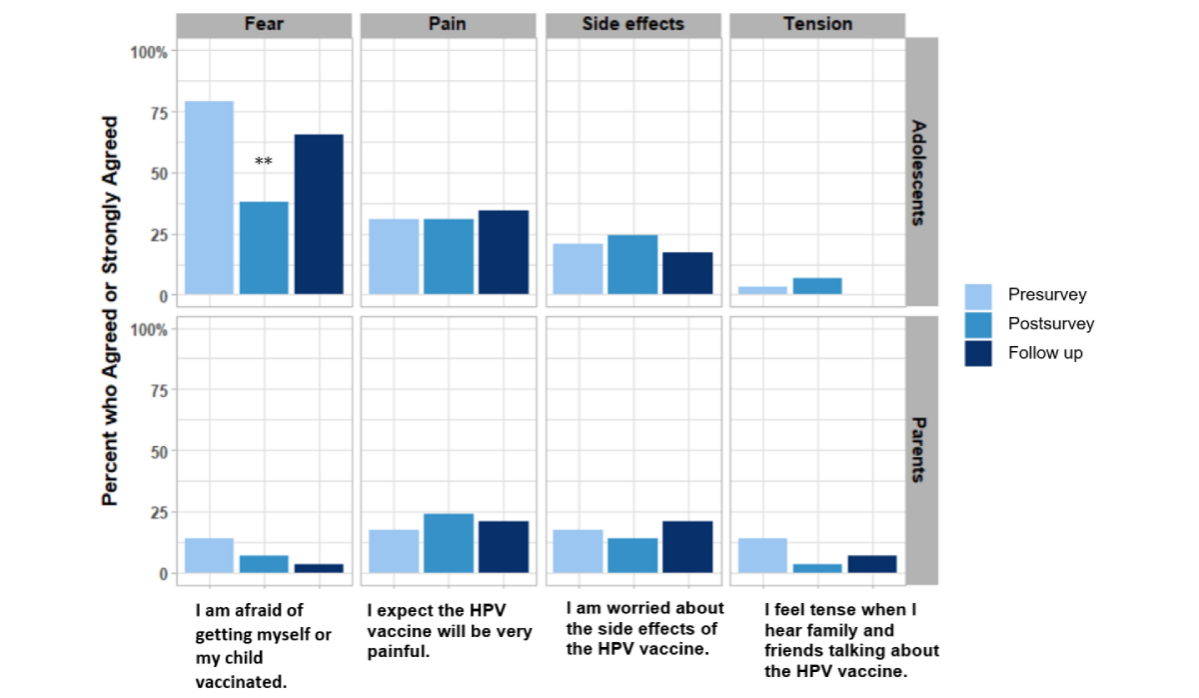
The percentages of participants who agreed or strongly agreed (4 or 5 on the Likert scale) to questions about HPV vaccine concerns were presented. McNemar test was used to assess the differences pre- versus post app and pre- versus follow-up. ** *P*<.01, * *P*<.05. HPV: human papillomavirus.

Figures 1 and 2 present the participants’ concerns about the potential impacts of HPV disease and HPV vaccine. There was a substantial increase in parents’ concerns about their teenagers developing penile cancer (*P*=.02) and genital warts (*P*=.02) from preintervention to 1 week post intervention (Figure 1). However, concerns were attenuated by follow-up, particularly regarding penile cancer, which dropped from 59% (17/29) to 41%(12/29) from post intervention to follow-up. There was also a substantial increase in adolescent girls’ concerns about cervical cancer (*P*=.02) and a significant increase in boys’ concerns about penile cancer (*P*=.02) 1 week post intervention, but similar to their parents, these concerns attenuated at follow-up.

Parents’ modest concerns, fears, and tensions about the vaccine did not change significantly throughout the study (Figure 2). In contrast, there was a substantial decrease in teenagers’ fear of vaccination (almost 80% at baseline) immediately after the intervention (*P*<.001). However, fear of vaccination increased at follow-up and was not significantly different from the baseline (*P*=.13).

### Parents’ Statements About Adolescents Obtaining the HPV Vaccine

Table 4 presents parents’ agreements with statements about obtaining the HPV vaccine. From baseline to follow-up, parents’ assessments that other parents were getting their children vaccinated (*P=*.03) and their feelings of support for getting the HPV vaccination (*P=*.02) significantly increased. In addition, there was a substantial increase in parents’ agreeing that providers thought that HPV vaccination was important (*P*=.002), they had enough information to make a decision about the HPV vaccination (*P*=.002), and they knew where to receive the HPV vaccination (*P*=.008).

**Table 4:** Change in parents’ statements about the human papillomavirus (HPV) vaccine.

Parent beliefs	Number of parents; Pre-HPV app, n (%)				Number of parents; Post-HPV app, n (%)				Number of parents; Follow-up, n (%)				*P* values^a^	
	Agree	Disagree	Not sure	Agree		Disagree	Not sure	Agree		Disagree	Not sure	Pre vs Post		Pre vs follow-up
Other parents in my community are getting their children the HPV shot or vaccine	8 (28)	4 (14)	17 (59)	11 (38)		6 (21)	12 (41)	14 (48)^b^		2 (7)	13 (45)	.55		.03
Most people who are important to me would support getting the HPV shot or vaccine for my child	14 (48)	5 (17)	10 (34)	18 (62)		7 (24)	4 (14)	22 (76)^b^		2 (7)	5 (17)	.42		.02
Doctors think it is very important that boys and girls get the HPV shot or vaccine	15 (52)	3 (10)	11 (38)	20 (69)		7 (24)	2 (7)	25 (86)^c^		2 (7)	2 (7)	.18		.002
I have enough information to make a decision about getting the HPV shot or vaccine for my child	14 (48)	4 (14)	11 (38)	20 (69)		7 (24)	2 (7)	26 (90)^c^		1 (3)	2 (7)	.18		.002
I know where my child can go to get the HPV vaccine or shot	19 (66)	6 (21)	4 (14)	20 (69)		6 (21)	3 (10)	27 (93)^c^		2 (7)	0 (0)	>.99		.008
It will be hard to find a provider or clinic where I can afford the HPV shot or vaccine for my child	5 (17)	18 (62)	6 (21)	4 (14)		21 (72)	4 (14)	4 (14)		23 (79)	2 (7)	>.99		>.99

^a^Reported *P* values are from McNemar exact test, which computes odds ratios that compare the number of parents who changed their opinion to agree after app use with those who changed their opinion to disagree or not sure after app use. Odds ratios (not shown) were significant only for pre–follow-up comparisons and not pre-post comparisons.

^b^*P*<.05.

^c^*P*<.01.

### Communicating and Seeking Information About HPV and HPV Vaccination

**Figure 3:**
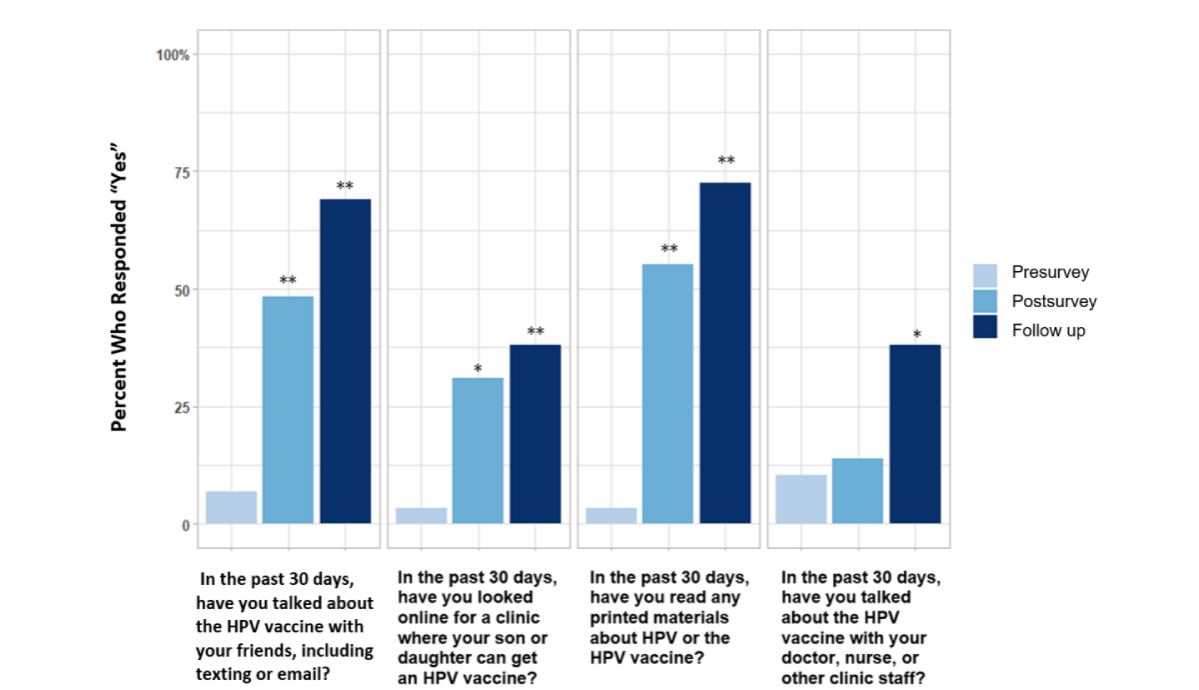
Parents’ seeking information and communicating about HPV and the HPV vaccine. The percentage of parents who responded “Yes” to questions about seeking information and communicating about HPV is presented. McNemar test was used to assess the differences pre- versus post app and pre- versus follow-up.** *P*<.01, * *P*<.05. HPV: human papillomavirus.

Parents reported substantial increases in communication and information seeking regarding HPV and HPV vaccination over the study period (Figure 3; Multimedia Appendix 1). From baseline to follow-up, there was an increase in parents who talked with friends (*P*<.001), read printed materials (*P<*.001), read web-based information (*P<*.001), communicated with their providers (*P=*.008), and searched on the web for a clinic to receive HPV vaccination (*P=*.007). Overall, teenagers did not show substantial changes in communication about HPV or the vaccine throughout the study period. However, there were substantial improvements in information-seeking from baseline to 1 week post intervention in reading printed materials (*P=*.005) and web-based information (*P=*.003; Multimedia Appendix 1).

### Decision-making About HPV Vaccination

Parents’ and adolescents’ agreement with decision-making statements regarding HPV vaccination are shown in Table 5. Almost all parents consistently agreed or strongly agreed that they were involved in deciding HPV vaccination uptake for their child. However, the HPV website improved collaborative decision-making between participants because there was a substantial increase in parents who agreed or strongly agreed that they included their child in the decision to receive the vaccine at 1 week post intervention (*P=*.002) and follow-up (*P=*.002). Approximately 40% (11/29) of adolescents agreed or strongly agreed that their parents decided that they would receive an HPV vaccination. However, like their parents, teenagers increasingly agreed that they jointly decided to receive an HPV shot, with *P=*.03 at 1 week post intervention and *P=*.02 at follow-up. Less than a quarter of parents and adolescents agreed that environmental and religious factors influenced their decision-making, although there was a substantial increase from baseline to follow-up on the influence of friends for parents (*P=*.02).

**Table 5:** Decision-making about getting the human papillomavirus (HPV) vaccination.

Percentage of people who strongly agree or agree		Pre-HPV app, n (%)	Post-HPV app, n (%)	Follow-up, n (%)	*P* values^a^	
					Pre vs Post	Pre vs follow-up
**Parents**						
	Parent made the decision	27 (93)	25 (86)	26 (90)	.48	.57
	Let their child make the decision	10 (34)	7 (24)	8 (28)	.28	.56
	Parent and child made the decision together	16 (55)	21 (72)^b^	22 (76)^b^	.002	.002
	Family and friends influenced decision	3 (10)	3 (10)	6 (21)^c^	.69	.02
	Religious beliefs influenced decision	3 (10)	3 (10)	5 (17)	.86	.33
**Adolescents**						
	My parents made the decision	11 (38)	12 (41)	11 (38)	.89	.81
	I made the decision myself	9 (31)	12 (41)^c^	11 (38)^c^	.03	.046
	I was involved in the decision	7 (24)	12 (41)^c^	14 (48)^c^	.03	.02
	My friends’ decisions influenced my decision	0 (0)	2 (7)	1 (3)	.62	.75
	Religious beliefs influenced decision	1 (3)	2 (7)	0 (0)	.23	.57
	I did not really think much about the decision	18 (62)	16 (55)	15 (52)	.20	.51

^a^Reported *P* values are from a paired *t* test (2-tailed) for a difference in mean Likert scores (not shown in Table). The percentages of strongly agree and agree responses (Likert scores of 4 and 5) are presented.

^b^*P*<.01.

^c^*P*<.05.

### Self-efficacy to Receive the HPV Vaccinations

Overall, parents had strong intentions to receive HPV vaccination for their children at baseline (Table 3), which increased at 1 week post intervention (*P*=.007) and follow-up (*P*=.05). The improvements were driven by an increased intention to talk with their child about getting the HPV vaccine, talking with their child’s physician or nurse, and initiating conversations with their child’s provider. Similar improvements occurred in the teenagers’ composite self-efficacy score, increasing at 1 week post intervention (*P*=.009) and follow-up (*P*=.007). The largest increase in adolescents’ self-efficacy from baseline to follow-up was knowing what to expect when receiving the HPV vaccination, although increases were seen across all 5 self-efficacy measures.

Although this pilot did not track receipt of the HPV vaccine, parents reported an increase in their children having had at least 1 HPV vaccine from 41% (12/29) at preintervention to 59% (17/29) at follow-up (*P*=.29). At follow-up, 59% (17/29) of adolescents also reported having received at least one HPV shot; unfortunately, adolescents were not asked this question preintervention. Many responses changed from “Not sure” to “Yes,” suggesting that the intervention increased parents’ knowledge of their child’s actual vaccination status.

### Usability and Acceptance of the HmongHPV Website

On the PSSUQ survey questions, parents reported high satisfaction with the HmongHPV website, with mean Likert scores ranging from 4.4 to 4.5 across all usability domains at 1 week post intervention and follow-up. The adolescents were slightly less satisfied, with a mean score ranging from 3.8 to 3.9 (Multimedia Appendix 1). There were no substantial changes in the parents or adolescents between the 2 posttime periods.

The qualitative interviews expanded beyond these quantitative usability scores. Generally, parents and adolescents found the website’s content informative and engaging; in particular, they enjoyed interactive quizzes and reminders. Parents reported more positive experiences with the website than did adolescents. For example, parents commonly reported that the website contained easy and accessible health information and had culturally appropriate content. In contrast, adolescents more often recounted that the website was too slow to load and did not contain visually appealing content and images. For example, they found images of reproductive organs with warts and cancers offensive. Both parents and teens recommended suggestions for improving the overall usability of the website: (1) allow users more control and flexibility when going through the educational modules (eg, skipping or viewing videos at a higher speed and rewatching old videos), (2) make messages and videos more concise, and (3) make the website more accessible (eg, providing an offline version and removing log-in features).

## Discussion

### Principal Findings

The pilot feasibility study showed that the HmongHPV website substantially improved and sustained parents’ and adolescents’ knowledge regarding HPV and the HPV vaccine and self-efficacy about obtaining the HPV vaccine over the 5-week study duration. In addition, parents’ and adolescents’ concerns about HPV-causing cancers increased, whereas concerns about the HPV vaccine causing harm decreased modestly. Parents’ health-seeking and communication behaviors increased, whereas parents’ and adolescents’ collaborative decision-making about receiving the HPV vaccination improved. In addition, the HmongHPV website was well received.

Theory-based eHealth studies on HPV adherence and uptake in Asian Americans are sparse. To our knowledge, this is the first study to create and evaluate a theory-based and CBPAR-informed Hmong-specific eHealth HPV vaccination educational intervention. Our SCT-informed intervention posits that personal (eg, knowledge, beliefs, and self-efficacy), behavioral (eg, decision-making and information-seeking), and environmental (eg, family, friend, provider, and community support; knowledge and access to clinical care) factors and their interactions influence an individual’s awareness and knowledge. With regard to personal factors, baseline knowledge of HPV and the HPV vaccine among participants was low, similar to other studies with Asian Americans whose baseline knowledge ranged from 19% to 65% [6,10,14,17,18,43,44]. However, the participants' knowledge increased substantially in the 5 weeks following the intervention. A web-based HPV intervention study with Chinese-American parents and adolescents reported similar findings, including increased knowledge among parents and adolescent participants [44]. However, in our study, knowledge improvement was more significant, at 38% (11/29) and 45% (13/29) for parents and adolescents, respectively. Zhu et al [44] concluded that an increase in knowledge leads to a 114% increase in the chance that a Chinese-American parent would *definitely* vaccinate their child, indicating that improvement in knowledge may translate to HPV vaccine uptake. Although intention does not directly correlate with actual behavior, previous studies [44] and findings from this study can improve the likelihood of behavioral change and HPV vaccine uptake.

Importantly, at the end of the study, parents (22/29, 76%) and adolescents (14/29, 48%) reported increased collective decision-making, significantly supporting their self-efficacy in obtaining vaccination. Zhu et al [44] found that 61% of Chinese parents would involve their child in decision-making; however, those who reported wanting to involve their children also reported lower intention scores. These findings suggest that parents are unsure of how their child would react to getting the vaccine and believe that their child might refuse, mainly contributing to their lack of HPV knowledge [44]. Our study found improved HPV and HPV vaccine knowledge among adolescents and parents, which may have contributed to increased joint decision-making for vaccine uptake. Clinical practices that support the participation of both parents and adolescents in the decision-making process might result in increased uptake and completion of the HPV vaccine series, especially if both parties are informed about HPV and the HPV vaccine. Zhu et al [44] found that the most statistically significant factors regarding vaccine intent were higher levels of HPV and HPV vaccine knowledge and doctors’ influence. Our study showed similar findings, with parents indicating information-seeking behaviors by talking with their friends and providers about HPV.

Parents and adolescents positively evaluated the website for usability, information quality, and interface quality while making recommendations for improvement. We attribute this reception to CBPAR practices, whereby Hmong-American community members on the research team and CAB served as equal partners during the intervention design, implementation, analyses, and interpretation. Parents rated the website more favorably than adolescents. Website expectations may be higher for adolescents than parents, as they are often more frequent internet users. As demonstrated in a disease prevention eHealth application with Vietnamese participants aged 15 to 24, older users perceived eHealth apps as more helpful and usable than younger participants [45].

The most relevant question is whether educational eHealth programs, such as HmongHPV, can improve HPV vaccination rates. However, this was not assessed in the pilot study. Although there was a slight increase in parents’ reporting that their children had an HPV shot during the study period, we could not attribute this to an actual increase in shots or improved knowledge of their child’s vaccination status throughout the study. In an eHealth study that used culturally tailored text messages for Korean-American women to obtain HPV vaccines, 30% of the participants initiated the HPV vaccine series within 3 months of the intervention and the intent to receive the vaccine within 1 year increased from 63% to 97% from pre- to post intervention [31]. Overall, this eHealth intervention improved HPV knowledge, self-efficacy, intention, and interactions with environmental support and may have increased vaccine uptake in a population that is historically known to have low vaccine levels and high rates of HPV-related cancers.

### Limitations

As a small pilot study with a nonprobability sample, the findings from this study may not be generalizable to the greater Hmong population and may contain self-report bias. Despite various recruitment approaches, enrolling participants was challenging and required a large incentive that may have led to additional self-report bias. In addition, the small sample size and short study duration limited the ability to examine potential predictors of increased HPV vaccine intentions or outcomes. A future randomized controlled study could be conducted to test the outcomes of HPV vaccination (eg, initiation and completion of vaccine series).

### Implications for Health Practice and Policy

Recent systematic reviews and studies on eHealth interventions to increase HPV vaccination adherence and uptake are promising, although none have addressed the US-based Asian communities, much less Hmong-American adolescents and their parents [37,46,47]. Most eHealth studies were reminder-based, education- and communication-oriented, or similar to our HmongHPV intervention, multicomponent strategies. This study demonstrated that CBPAR-informed and theory-based eHealth applications are a feasible method for disseminating HPV information to Hmong-American adolescents and their parents. These digital interventions are rising in popularity, and leveraging this platform can be beneficial in addressing issues in hard-to-reach populations to increase HPV vaccination rates. Improved HPV and HPV vaccine awareness and knowledge, health literacy, care access, and addressing sociocultural beliefs may increase vaccine uptake in many Asian-American subpopulations. eHealth interventions provide opportunities to eliminate some of these barriers by providing widely available and culturally tailored HPV educational messages and facilitating access to providers.

This study’s findings show that within the Hmong-American population, an eHealth HPV intervention created in partnership with community members can improve knowledge, self-efficacy, decision-making, and information-seeking behaviors regarding HPV and the HPV vaccine. The *Hmong Promoting Vaccines* website was also well received by adolescents and adults, suggesting the potential for broader application beyond this study group. With the current COVID-19 pandemic, the use of eHealth applications can ensure that safety precautions are adhered to and may become the preferred method of implementation of health education materials and interventions in the future. Further research is needed to determine whether the current eHealth web-based education is indeed useful in the uptake and completion of the HPV vaccine series, the key factors contributing to vaccine acceptance, and how best to disseminate and implement such eHealth interventions sustainably. Future studies should continue to offer interventions informed by the CBPAR principles to address communities’ specific issues in receiving preventive health care services.

## Appendix

Multimedia Appendix 1 Vaccine concerns.

## Abbreviations

CAB: community advisory board
CBPAR: community-based participatory action research
HPV: human papillomavirus
PSSUQ: Poststudy System Usability Questionnaire
SCT: Social Cognitive Theory

## Data Availability

The data sets generated and analyzed during this study are available from the corresponding author upon reasonable request.
