# Supplementary material for: Evaluating Human Papillomavirus eHealth in Hmong Adolescents to Promote Vaccinations: Pilot Feasibility Study
Source: JMIR Form Res. 2023 Jun 20;7:e38388. doi: 10.2196/38388 (PMC10337404; doi:10.2196/38388)
Supplement: Multimedia Appendix 1 [file formative_v7i1e38388_app1.docx]

**Multimedia Appendix 1. Vaccine concerns.**

**Supplementary Table 1a. Change in parents’ HPV knowledge**

|  | ***Pre-HPV App*** | | | ***Post-HPV App*** | | | ***Follow-up*** | | |
| --- | --- | --- | --- | --- | --- | --- | --- | --- | --- |
| **Disease Knowledge**  **(# of parents)** | **True** | **False** | **Do not know** | **True** | **False** | **Do not know** | **True** | **False** | **Do not know** |
| **1**. **HPV infection is different than HIV infection.** | 18 | 4 | 7 | 25 | 1 | 3 | 24 | 3 | 2 |
| **2. HPV infection is very**  **rare.** | 5 | 16 | 8 | 3 | 21 | 5 | 5 | 22 | 2 |
| **3**. **Males cannot get HPV.** | 2 | 21 | 6 | 1 | 24 | 4 | 4 | 23 | 2 |
| **4. HPV can be caught through someone sneezing or coughing on you.** | 4 | 9 | 16 | 2 | 24 | 3 | 4 | 23 | 2 |
| **5. HPV can be caught by shaking hands with someone.** | 2 | 15 | 12 | 3 | 24 | 2 | 1 | 25 | 3 |
| **6. HPV can be caught through sexual contact or having sex with someone.** | 19 | 0 | 10 | 27 | 0 | 2 | 28 | 0 | 1 |
| **7. Most people with an HPV infection know they have it.** | 4 | 14 | 11 | 3 | 24 | 2 | 4 | 22 | 3 |
| **8. Most people with an HPV infection have symptoms.** | 6 | 9 | 14 | 4 | 22 | 3 | 5 | 21 | 3 |
| **9. HPV can cause cancer of the cervix.** | 16 | 1 | 12 | 28 | 0 | 1 | 28 | 1 | 0 |
| **10. HPV can cause cancer of the penis.** | 12 | 1 | 16 | 26 | 0 | 3 | 25 | 2 | 2 |
| **11. HPV can cause lung cancer.** | 4 | 9 | 16 | 6 | 19 | 4 | 6 | 19 | 4 |
| **12. HPV can cause genital warts (i.e., warts on private body parts).** | 12 | 2 | 15 | 27 | 0 | 2 | 26 | 1 | 2 |
| **13. Medicines are needed to treat HPV infection.** | 14 | 3 | 12 | 21 | 5 | 3 | 21 | 6 | 2 |
| **14. HPV infection will go away on it's own, with no treatment.** | 2 | 15 | 12 | 3 | 23 | 3 | 4 | 22 | 3 |

Parent answers to the fourteen HPV knowledge questions are presented. Gray boxes are the correct answers.

**Supplementary Table 1b. Change in adolescents’ HPV knowledge**

|  | ***Pre-HPV App*** | | | ***Post-HPV App*** | | | ***Follow-up*** | | |
| --- | --- | --- | --- | --- | --- | --- | --- | --- | --- |
| **Disease Knowledge**  **(# of adolescents)** | **True** | **False** | **Do not know** | **True** | **False** | **Do not know** | **True** | **False** | **Do not know** |
| **1**. **HPV infection is different than HIV infection.** | 9 | 0 | 20 | 15 | 4 | 10 | 19 | 1 | 9 |
| **2. HPV infection is very**  **rare.** | 2 | 10 | 17 | 3 | 20 | 6 | 2 | 19 | 8 |
| **3**. **Males cannot get HPV.** | 0 | 18 | 11 | 1 | 26 | 2 | 0 | 27 | 2 |
| **4. HPV can be caught through someone sneezing or coughing on you.** | 3 | 13 | 13 | 2 | 23 | 4 | 6 | 20 | 3 |
| **5. HPV can be caught by shaking hands with someone.** | 1 | 17 | 11 | 6 | 19 | 4 | 5 | 20 | 4 |
| **6. HPV can be caught through sexual contact or having sex with someone.** | 17 | 2 | 10 | 27 | 0 | 2 | 28 | 0 | 1 |
| **7. Most people with an HPV infection know they have it.** | 3 | 6 | 20 | 3 | 24 | 2 | 3 | 20 | 6 |
| **8. Most people with an HPV infection have symptoms.** | 14 | 2 | 13 | 11 | 13 | 5 | 13 | 11 | 5 |
| **9. HPV can cause cancer of the cervix.** | 6 | 2 | 21 | 26 | 1 | 2 | 26 | 1 | 2 |
| **10. HPV can cause cancer of the penis.** | 4 | 1 | 24 | 24 | 1 | 4 | 26 | 0 | 3 |
| **11. HPV can cause lung cancer.** | 1 | 5 | 23 | 7 | 15 | 7 | 5 | 14 | 10 |
| **12. HPV can cause genital warts (i.e., warts on private body parts).** | 9 | 2 | 18 | 28 | 0 | 1 | 27 | 0 | 2 |
| **13. Medicines are needed to treat HPV infection.** | 16 | 1 | 12 | 23 | 1 | 5 | 19 | 4 | 6 |
| **14. HPV infection will go away on its own, with no treatment.** | 1 | 12 | 16 | 9 | 15 | 5 | 7 | 13 | 9 |

Child answers to the fourteen HPV knowledge questions are presented. Gray boxes are the correct answers.

**Supplementary Table 2a. Change in parents’ HPV vaccine knowledge**

|  | ***Pre-HPV App*** | | | ***Post-HPV App*** | | | ***Follow-up*** | | |
| --- | --- | --- | --- | --- | --- | --- | --- | --- | --- |
| **Vaccine Knowledge**  **(# of parents)** | **True** | **False** | **Do not know** | **True** | **False** | **Do not know** | **True** | **False** | **Do not know** |
| **1. Shots/vaccines are necessary to protect the health of children your son or daughter's age.** | 24 | 2 | 3 | 28 | 1 | 0 | 26 | 0 | 3 |
| **2. Shots/vaccines do a good job preventing the diseases they are intended to prevent.** | 23 | 0 | 6 | 28 | 0 | 1 | 27 | 0 | 2 |
| **3.Shots/vaccines are safe.** | 19 | 3 | 7 | 24 | 2 | 3 | 27 | 0 | 2 |
| **4. The HPV shot/vaccine keeps people from having serious health problems.** | 21 | 2 | 6 | 25 | 3 | 1 | 24 | 2 | 3 |
| **5. The HPV shot/vaccine is not safe.** | 3 | 18 | 8 | 1 | 26 | 2 | 2 | 25 | 2 |
| **6. Girls 11 to 17 years old should get the HPV shot/vaccine.** | 18 | 1 | 10 | 23 | 3 | 3 | 25 | 1 | 3 |
| **7. Boys 11 to 17 years old do not need the HPV shot/vaccine.** | 5 | 14 | 10 | 10 | 16 | 3 | 7 | 18 | 4 |
| **8. HPV vaccination is only one shot.** | 3 | 15 | 11 | 4 | 22 | 3 | 4 | 21 | 4 |
| **9. The HPV shot/vaccine protects against cervical cancer.** | 11 | 3 | 15 | 28 | 0 | 1 | 25 | 1 | 3 |
| **10. The HPV vaccine/shot is only for people who are having sex now.** | 2 | 15 | 12 | 1 | 25 | 3 | 3 | 24 | 2 |
| **11. The HPV vaccine/shot is best for people who have never had sex.** | 6 | 12 | 11 | 10 | 16 | 3 | 14 | 13 | 2 |
| **12. People who have had sex already should not get the HPV shot/vaccine.** | 2 | 14 | 13 | 0 | 26 | 3 | 2 | 26 | 1 |

Parent answers to the twelve HPV vaccine knowledge questions are presented. Gray boxes are the correct answers.

**Supplementary Table 2b. Change in adolescents’ HPV knowledge**

|  | ***Pre-HPV App*** | | | ***Post-HPV App*** | | | ***Follow-up*** | | |
| --- | --- | --- | --- | --- | --- | --- | --- | --- | --- |
| **Vaccine Knowledge**  **(# of adolescents)** | **True** | **False** | **Do not know** | **True** | **False** | **Do not know** | **True** | **False** | **Do not know** |
| **1. Shots/vaccines are necessary to protect my health.** | 24 | 0 | 5 | 26 | 1 | 2 | 24 | 1 | 4 |
| **2. Shots/vaccines do a good job preventing the diseases they are intended to prevent.** | 23 | 0 | 6 | 25 | 0 | 4 | 25 | 0 | 4 |
| **3.Shots/vaccines are safe.** | 21 | 0 | 8 | 25 | 0 | 4 | 25 | 0 | 4 |
| **4. The HPV shot/vaccine keeps people from having serious health problems.** | 17 | 1 | 11 | 22 | 4 | 3 | 25 | 1 | 3 |
| **5. The HPV shot/vaccine is not safe.** | 0 | 17 | 12 | 2 | 24 | 3 | 0 | 24 | 5 |
| **6. Girls 11 to 17 years old should get the HPV shot/vaccine.** | 13 | 1 | 15 | 25 | 0 | 4 | 23 | 2 | 4 |
| **7. Boys 11 to 17 years old do not need the HPV shot/vaccine.** | 3 | 12 | 14 | 16 | 9 | 4 | 14 | 11 | 4 |
| **8. HPV vaccination is only one shot.** | 1 | 8 | 20 | 1 | 22 | 6 | 2 | 19 | 8 |
| **9. The HPV shot/vaccine protects against cervical cancer.** | 5 | 3 | 21 | 23 | 0 | 5 | 24 | 0 | 5 |
| **10. The HPV vaccine/shot is only for people who are having sex now.** | 0 | 12 | 17 | 1 | 20 | 8 | 1 | 23 | 5 |
| **11. The HPV vaccine/shot is best for people who have never had sex.** | 1 | 7 | 21 | 5 | 10 | 14 | 8 | 13 | 8 |
| **12. People who have had sex already should not get the HPV shot/vaccine.** | 1 | 11 | 17 | 1 | 21 | 7 | 1 | 22 | 6 |

Child answers to the twelve HPV vaccine knowledge questions are presented. Gray boxes are the correct answers.

**Supplementary Table 3. Communication about HPV and the HPV vaccine**

| Communication about HPV and the vaccine  # who responded yes (%) | *Pre-HPV App* | *Post-HPV App* | *Follow-up* |
| --- | --- | --- | --- |
|  | **Parents** |  |  |
| In the past 30 days, have you talked about the HPV shot/vaccine with your friends, including texting or e-mail? | 2 (7%) | **14 (48%) | **20 (69%) |
| In the past 30 days, have you talked about the HPV shot/vaccine with your doctor, nurse, or other clinic staff? | 3 (10%) | 4 (14%) | *11 (38%) |
| In the past 30 days, have you read any printed materials about HPV or the HPV shot/vaccine? | 1 (3%) | **16 (55%) | **21 (72%) |
| In the last 30 days, have you read anything about HPV or the HPV shot/vaccine on-line (i.e., internet or the world wide web)? | 2 (7%) | **20 (69%) | **21 (72%) |
| In the past 30 days, have you looked online for a clinic or provider where your son or daughter can get the HPV shot/vaccine? | 1 (3%) | *9 (31%) | **11 (38%) |
| In the last 30 days, have you used any Apps about HPV or the HPV shot/vaccine? | 1 (3%) | **11 (38%) | **14 (48%) |
| Adolescents | | | |
| In the past 30 days, have you talked about the HPV shot/vaccine with your friends, including texting or e-mail? | 2 (7%) | 5 (17%) | 4 (14%) |
| In the past 30 days, have you talked about the HPV shot/vaccine with your doctor, nurse, or other clinic staff? | 1 (3%) | 2 (10%) | 4 (14%) |
| In the past 30 days, have you read any printed materials about HPV or the HPV shot/vaccine? | 1 (3%) | **13 (45%) | 5 (17%) |
| In the last 30 days, have you read anything about HPV or the HPV shot/vaccine on-line (i.e., internet or the world wide web)? | 1 (3%) | **18 (62%) | **9 (31%) |
| In the past 30 days, have you looked online for a clinic or provider where you can get the HPV shot/vaccine? | 0 (0%) | 3 (10%) | 3 (10%) |
| In the last 30 days, have you used any Apps about HPV or the HPV shot/vaccine? | 0 (0%) | 3 (10%) | 1 (3%) |

The table shows the number and percentage of parents and children who responded “yes” to questions about communication.

Since most comparisons had less than 25 discordant pairs, exact McNamar tests were used.

**Supplementary Table 4a. HPV web application post-survey system user**

**Questionnaire: System usefulness**

| Parents | | | | Adolescents | | | |
| --- | --- | --- | --- | --- | --- | --- | --- |
| System Usefulness  (mean Likert score) | ***Post-HPV App*** | ***Follow-up*** | ***Post vs. Follow-up*** | | ***Post-HPV App*** | ***Follow-up*** | ***Post vs. Follow-up*** |
| Overall, I am satisfied with how easy it is to use the Hmong HPV App | 4.55 | 4.48 | -0.07  (-0.36, 0.22) | | 3.93 | 3.97 | 0.03  (-0.39, 0.46) |
| I was able to finish all the stories and questions included as part of the Hmong HPV App | 4.58 | 4.58 | 0  (-0.23, 0.23) | | 4.06 | 3.89 | -0.17  (-0.68, 0.34) |
| I felt comfortable using the Hmong HPV App | 4.51 | 4.44 | -0.07  (-0.29, 0.16) | | 3.97 | 3.89 | -0.07  (-0.5, 0.36) |
| It was easy to learn to use the Hmong HPV App | 4.51 | 4.37 | -0.14  (-0.45, 0.18) | | 4.00 | 4.03 | 0.03  (-0.38, 0.45) |
| System usefulness score | 4.54 | 4.47 | -0.07  (-0.31, 0.17) | | 3.99 | 3.95 | -0.04  (-0.44, 0.35) |

Mean Likert responses are shown for each question. Each participant had an overall system usefulness score which was the average of the 4 individual questions. Paired t-tests and confidence intervals are shown comparing opinions immediately after using the HPV app and during follow-up.

**Supplementary Table 4b. HPV web application post-survey system user**

**Questionnaire: Information quality**

| Parents | | | | Adolescents | | |
| --- | --- | --- | --- | --- | --- | --- |
| Information quality  (mean Likert score) | ***Post-HPV App*** | ***Follow-up*** | ***Post vs. Follow-up*** | ***Post-HPV App*** | ***Follow-up*** | ***Post vs. Follow-up*** |
| When I made a mistake using the Hmong HPV App, I could fix it quickly and easily | 4.10 | 4.34 | 0.24  (-0.11, 0.59) | 3.83 | 3.78 | -0.07  (-0.5, 0.36) |
| It was easy to find the information I needed | 4.21 | 4.48 | 0.28  (-0.01, 0.56) | 3.93 | 4.00 | 0.07  (-0.35, 0.49) |
| The information in the Hmong HPV App was easy to understand | 4.45 | 4.45 | 0  (-0.23, 0.23) | 4.07 | 4.0 | 0  (-0.37, 0.37) |
| I learned a lot about HPV from the Hmong HPV App | 4.55 | 4.55 | 0  (-0.25, 0.25) | 4.07 | 3.97 | -0.1  (-0.54, 0.33) |
| I learned a lot about the HPV shot/vaccine from the Hmong HPV App | 4.48 | 4.58 | 0.1  (-0.13, 0.34) | 4.00 | 3.79 | -0.21  (-0.71, 0.3) |
| Finding information in the Hmong HPV App was clear | 4.45 | 4.41 | -0.03  (-0.25, 0.18) | 4.00 | 4.03 | 0.03  (-0.37, 0.43) |
| Information quality score | 4.37 | 4.47 | 0.10  (-0.11, 0.30) | 3.98 | 3.94 | -0.05  (-0.43, 0.35) |

Mean Likert responses are shown for each question about information quality. Each participant had an overall information quality score which was the average of the 6 individual questions. Paired t-tests and confidence intervals are shown comparing opinions immediately after using the HPV app and during follow-up.

**Supplementary Table 4c. HPV web application post-survey system user**

**Questionnaire: Interface quality**

| Parents Adolescents | | | | | |  |
| --- | --- | --- | --- | --- | --- | --- |
| Interface quality and overall satisfaction  (mean Likert score) | ***Post-HPV App*** | ***Follow-up*** | ***Post vs. Follow-up*** | ***Post-HPV App*** | ***Follow-up*** | ***Post vs. Follow-up*** |
| The HPV App screens, questions, and texts were pleasant | 4.41 | 4.41 | 0  (-0.2, 0.2) | 3.93 | 3.86 | -0.07  (-0.39, 0.25) |
| I am satisfied with how the Hmong HPV App works with my phone, and/or computer | 4.45 | 4.55 | 0.1  (-0.11, 0.32) | 3.86 | 3.97 | 0.1  (-0.32, 0.53) |
| I like what was in the phone text reminders | 4.21 | 4.21 | 0  (-0.25, 0.25) | 3.79 | 3.79 | 0  (-0.38, 0.38) |
| I like how often I get the Hmong HPV App text messages | 4.07 | 4.07 | 0  (-0.37, 0.37) | 3.52 | 3.38 | -0.14  (-0.47, 0.19) |
| The health navigator services are helpful | 4.21 | 4.28 | 0.07  (-0.22, 0.36) | 3.72 | 3.75 | 0.03  (-0.41, 0.48) |
| I like using the Hmong HPV App | 4.31 | 4.34 | 0.03  (-0.22, 0.29) | 3.72 | 3.76 | 0.04  (-0.39, 0.46) |
| I would recommend the Hmong HPV App to family and friends | 4.31 | 4.34 | 0.03  (-0.26, 0.33) | 3.69 | 3.72 | 0.03  (-0.39, 0.46) |
| The Hmong HPV App has everything I expected it to have | 4.17 | 4.28 | 0.1  (-0.17, 0.38) | 3.48 | 3.69 | 0.21  (-0.2, 0.62) |
| Interface quality score | 4.34 | 4.41 | 0.07  (-0.12, 0.26) | 3.75 | 3.84 | 0.08  (-0.25, 0.42) |

Mean Likert responses are shown for each question about interface quality. T-tests and confidence intervals are shown comparing opinions immediately after using the HPV app and during follow-up. ^a^ These measures used to calculate the ‘interface quality’ subscore.
